# Screening for atrial fibrillation during automated blood pressure measurement among patients admitted to internal medicine ward

**DOI:** 10.1007/s11739-021-02691-2

**Published:** 2021-03-20

**Authors:** Giacomo Pucci, Edoardo Santoni, Valeria Bisogni, Camilla Calandri, Alberto Cerasari, Irene Dominioni, Leandro Sanesi, Marco D’Abbondanza, Vito Veca, Gaetano Vaudo

**Affiliations:** 1 Department of Medicine and Surgery, University of Perugia, Perugia, Italy; 2 Unit of Internal Medicine, Terni University Hospital, Terni, Italy; 3 Department of Medicine and Surgery, Unit of Internal Medicine, Terni University Hospital, University of Perugia, Piazzale Tristano Di Joannuccio, 1, T05100 Terni, Italy

**Keywords:** Atrial fibrillation, Blood pressure, Screening, Cardiovascular prevention, Internal medicine

## Abstract

Atrial fibrillation (AF), the commonest sustained cardiac arrhythmia affecting the adult population, is often casually discovered among hospitalized people. AF onset is indeed triggered by several clinical conditions such as acute inflammatory states, infections, and electrolyte disturbance, frequently occurring during the hospitalization. We aimed to evaluate whether systematic AF screening, performed through an automated oscillometric blood pressure (BP) device (Microlife WatchBP Office AFIB, Microlife AG, Switzerland), is effective for detecting AF episodes in subjects admitted to an Internal Medicine ward. 163 patients consecutively hospitalized at the Unit of Internal Medicine of the “Santa Maria” Terni University Hospital between November 2019 and January 2020 (mean age ± standard deviation: 77 ± 14 years, men proportion: 40%) were examined. Simultaneously with BP measurement and AF screening, a standard 12-lead electrocardiogram (ECG) was performed in all subjects. AF was diagnosed by ECG in 29 patients (18%). AF screening showed overall 86% sensitivity and 96% specificity. False negatives (*n* = 4) had RR-interval coefficient of variation lower than true positives (*n* = 25, *p* < 0.01), suggesting a regular ventricular rhythm during AF. The repeated evaluation substantially confirmed the same level of agreement. AF screening was positive in all patients with new-onset AF (*n* = 6, 100%). Systematic AF screening in patients admitted to Internal Medicine wards, performed using the Microlife WatchBP Office AFIB, is feasible and effective. The opportunity to implement such technology in daily routine clinical practice to prevent undiagnosed AF episodes in hospitalized patients should be the subject of further research.

## Introduction

Atrial fibrillation (AF), the commonest sustained cardiac arrhythmia affecting the adult population, has a significative adverse impact on patient management and on the healthcare system. The overall risk of death and disability attributable to AF is related to stroke susceptibility, symptoms severity, the severity of AF burden and the cardiac substrate [1]. Such risk could be significantly reduced by timing diagnosis and appropriate pharmacological management. AF occurs asymptomatically in about 10–20% of patients [2] and the prevalence of asymptomatic AF is estimated to be 0.5–1% of the general population, with a dramatic increment with aging and it is, therefore, projected to increase in future years [3]. This clinical condition portends an increased risk of ischemic cardio-embolic stroke because of the lack of appropriate anticoagulation in people with high thrombo-embolic risk. It is estimated that 1 out of 10 cardio-embolic strokes are related to asymptomatic AF [4].

The AF onset could be triggered by a number of cardiac and non-cardiac pathogenic conditions, often occurring during hospitalization. These are sepsis, acute inflammatory diseases, pneumonia, cardiac and non-cardiac surgery, electrolytic disturbance, pulmonary embolism, acute heart failure, and thyrotoxicosis [5–7]. Moreover, frequently, the onset of AF complicates the clinical course of hospitalized patients admitted to Internal Medicine wards. Indeed, such patients have been found to have a higher rate of new-onset AF during the entire length of hospital stay [8, 9], including asymptomatic episodes. This is of relevance, given that new-onset AF prolongs the course of hospitalization, and it is independently associated with adverse prognosis [10].

Current guidelines recommend a proper AF screening in patients with systemic arterial hypertension, obstructive sleep apneas, or aged ≥ 65 years old [1]. All these conditions are frequently observed in subjects hospitalized at Internal Medicine wards, but a systematic AF screening in these patients, at present, is not performed, even though many randomized-controlled trials realized to confirm and quantify the potential benefits of AF screening in different clinical contexts are ongoing, along with many initiatives aimed at implementing the AF search culture in clinical practice [11]. Some studies demonstrated that, compared to pulse palpation, novel screening methodologies for AF identification, such as portable electrocardiography (ECG) monitors, ECG patches, smartwatches, and blood pressure (BP) measurement devices, might help in reducing undiagnosed AF cases, especially asymptomatic AF episodes, even if performed at a single time point and irrespective of the clinical context [2]. These devices are recommended as part of appropriate screening of AF in patients with high risk, and are effective in increasing diagnosis, although with sub-optimal performance [12]. BP monitor devices are particularly suitable for routine daily hospital management, because of their ease of use and the simultaneous detection of BP, a fundamental vital sign, along with AF screening.

The aim of the present study is to evaluate the accuracy of AF episodes detection of the Microlife WatchBP Office AFIB device, an instrument that has been recommended for AF screening in general population [13], in consecutive patients hospitalized at Internal Medicine wards.

## Methods

### Study population

The study was carried out in the Unit of Internal Medicine of “Santa Maria” University Hospital of Terni, Italy, a third-level general hospital covering an estimated population of about 230.000 inhabitants of the southern part of the Umbria region. The Unit is equipped with 24 beds with a yearly number of about 1500 hospitalizations.

The primary outcome of this study was to determine the sensitivity, specificity, positive predictive value (PPV), and negative predictive value (NPV) of AF screening performed in all hospitalized patients at the Internal Medicine ward during routine clinical practice using the Microlife WatchBP Office AFIB device (Microlife AG, Widnau, Switzerland). Secondary objectives were to evaluate the reproducibility of AF screening by repeated measurements, to evaluate the performance of AF screening in subjects aged ≥ 65 years, and to assess the effectiveness of screening for new-onset AF episodes recorded during the hospitalization period.

Hospitalized patients at the Unit of Internal Medicine between November 2019 and January 2020 were all invited to participate to the study. All procedures were detailed to the patients and to their caregivers, if needed. Patients were excluded if they: (1) wore a pacemaker, or (2) had an upper arm circumference < 22 or > 42 cm, or (3) were unable to give informed consent to participate at the study. The ethical approval for study conduction was obtained by internal Ethic Committee.

### Procedures

Measurements were performed in the morning in fasting, between days 1 and 3 of admission, with the patient comfortably laying supine on his/her bed after 5 min of rest. The operators (CC and ES) were not aware of the AF current or previous patients’ history before the procedure. After completing the exam, data about clinical and anthropometric variables, plus other relevant information including detailed information about past AF history were collected by medical personnel through individual interviews and consultation of past medical records. The main routine laboratory findings determined at hospital admission were derived from electronic medical records. New-onset AF was defined if sinus rhythm was previously diagnosed at the 12-lead ECG recorded at admission (included at the emergency department), and if the patient was not aware of his/her AF status.

After placing the brachial cuff on the non-dominant arm, the patient was left alone in the room and asked to rest for at least 10 min. Speaking was not allowed from the beginning to the end of the entire procedure. BP was measured by a brachial validated oscillometric device, the Microlife WatchBP Office AFIB. It is equipped with a brachial cuff suitable for an upper arm circumference included between 22 and 42 cm, and it has been previously validated for oscillometric automated BP measurements [14]. This device also allows the analysis of pulse irregularities during cuff deflation [15] by a specific algorithm after excluding incidental arrhythmias caused by premature beats. According to device’s user manual, it was set on automatic 3 sequential readings mode and AF screening was performed on 3 BP measurements automatically taken in sequence with an interval of 15 s each. At the end of the third measurement, BP and HR resulting from the average of the three readings were recorded for subsequent analysis and AF screening was evaluated as positive if a dedicated symbol (Afib) appeared on the display of the BP monitor.

Simultaneously with BP automated measurement, a 12-lead standard ECG was recorded and printed for subsequent analysis performed by a cardiologist (GP). The R–R intervals between consecutive beats marked as those recorded during cuff deflation were recorded to calculate the R–R coefficient of variation (RR-CV), as the ratio of the standard deviation to the mean value. The entire procedure, along with 12-lead ECG, was repeated twice in each patient irrespective of the results of the screening procedure.

### Statistical analysis

All continuous variables were expressed as mean ± standard deviation (SD) if normally distributed, or as median and interquartile range (IQR) if non-normally distributed, or as relative (%) frequencies in case of categorical variables. The assumption of satisfactory normal distribution was tested for all the examined variables by the Kolmogorov–Smirnov *Z* test. Differences between subjects screened positive for AF (AF patients) and negative for AF (non-AF patients) were evaluated through the Student’s *T*-test for independent samples or Mann–Whitney test, when applicable. Cohen’s kappa coefficient (*κ*) was calculated to assess and rate the significance and the level of agreement between the screening test and the gold standard for AF diagnosis, with *κ* values being interpreted according to the Landis and Koch scale [16]. Sensitivity was calculated as the ratio between true positives/all AF rhythms diagnosed at the ECG and specificity as the ratio between true negatives/all non-AF rhythms diagnosed at the ECG. PPV and NPV were calculated as the ratio of true positive/negative *vs* all positive/negative at the AF screening. The comparison of sensitivity and specificity with repeated testing was assessed through the calculation of the McNemar *χ*^2^ test with continuity correction. Statistical analysis was performed using IBM SPSS 24 (IBM Corporation). Statistical significance was set at *p* < 0.05.

## Results

Between November 2019 and January 2020, a total of 181 patients consecutively admitted to the Unit of Internal Medicine, representing the 12% of the yearly number of admissions, were screened for study enrolment. Eighteen patients (10% of the study population) were excluded according to the exclusion criteria above mentioned. The remaining patients (*n* = 163) successfully completed the study protocol and entered the subsequent analysis.

AF was diagnosed by ECG in 29 patients, corresponding to the 18% of the total population under the evaluation. In one case, the ECG recorded supraventricular tachycardia with regular ventricular response that, for such reason, was classified among non-AF rhythms. All the left non-AF were found to be sinus rhythms. AF was newly diagnosed in 6 patients, corresponding to the 4% of the entire enrolled population and to the 21% of all AF episodes. All other people with AF were aware of their clinical condition. Nine patients who reported a positive history of AF, indeed had sinus rhythm during evaluation.

In Table 1, the main characteristics of the study population were detailed by AF diagnosis at the ECG. Subjects with AF were older than subjects with non-AF rhythms (86 ± 6 *vs* 75 ± 15 years, *p* < 0.001) and more frequently presented a positive history of AF (79 *vs* 7%, *p* < 0.001). Subjects with AF more frequently showed HR higher than 100 bpm (17 *vs* 5%, *p* = 0.03) and systolic BP lower than 100 mmHg (14 *vs* 4%, *p* = 0.03). Moreover, a diagnosis of heart failure (*p* < 0.01) and previous CV events, such as coronary artery diseases, cerebrovascular accidents, and peripheral arterial disease (*p* < 0.05), was more often found among subjects with AF than among those with non-AF rhythms. The two groups did not differ in terms of sex prevalence, average BP and HR, and upper arm circumference.

**Table 1:** Main clinical and laboratory characteristics of the study population by AF status at the 12-lead ECG

	Total	AF	Non-AF	*p*
*n*. (%)	163	29 (18)	134 (82)	
Age, years	77 ± 14	86 ± 6	75 ± 15	< 0.001
Male sex, %	40	48	39	0.45
Upper arm circumference, cm	28 ± 3	28 ± 2	28 ± 3	0.52
Previous CV event, %	23	36	20	< 0.05
Heart failure, %	33	72	24	< 0.01
Chronic kidney disease, %	18	18	19	0.79
Type 2 diabetes mellitus, %	29	31	21	0.29
COPD, %	14	21	6	0.09
Known AF, n. (%)	32 (20)	23 (79)	9 (7)	< 0.001
New onset AF, %	6 (4)	6 (21)	–	–
Heart rate (by ECG) > 100 bpm, n. (%)	11 (7)	5 (17)	6 (5)	0.01
Systolic BP < 100 mmHg, n. (%)	9 (6)	4 (14)	5 (4)	0.03
Serum creatinine, mg/dL	1.3 ± 0.9	1.3 ± 1.0	1.3 ± 0.5	0.76
Serum potassium, mmol/L	4.1 ± 0.4	4.0 ± 0.5	4.1 ± 0.5	0.49
Hemoglobin, g/dL	11.4 ± 2.5	11.1 ± 2.0	11.4 ± 2.6	0.42
Fasting serum glucose, mg/dL	151 ± 61	130 ± 34	146 ± 56	0.14

Data are represented as mean ± SD

*AF* atrial fibrillation, *BP* blood pressure, *ECG* electrocardiogram, *CV* cardiovascular, *COPD* chronic obstructive pulmonary disease

AF rhythm was correctly detected by WatchBP device in 25 out of 29 patients (sensitivity 86%, Table 2); whereas in 5 patients, AF signaling by WatchBP device was not confirmed at the ECG evaluation (specificity 96%). The overall level of agreement was almost perfect according to Cohen kappa’s coefficient (0.81, *p* < 0.001). PPV and NPV were 83 and 97%, respectively. Patients with false-negative results at the WatchBP for AF signaling (*n* = 4) showed an RR-CV between 10 consecutive heartbeats at the ECG significantly lower than subjects with AF at the ECG and correctly identified by the WatchBP (0.09 ± 0.02 *vs* 0.19 ± 0.06, *p* < 0.01). No other significant differences were found between false negatives and true positives, as well as between false positives (*n* = 5) and true negatives regarding the distribution of the other examined variables (Table 3).

**Table 2:** Performance of the Microlife WatchBP Office AFIB for the screening of atrial fibrillation (AF) compared with standard 12-lead electrogram (ECG)

	AF ECG	Non-AF ECG	Total
AF WatchBP	25	5	30
Non-AF WatchBP	4	129	133
Total	29	134	163

*AF* atrial fibrillation, *Non-AF* rhythm other than atrial fibrillation

**Table 3:** Clinical and electrocardiographic characteristics of patients according to AF screening status

	True positives	False negatives	*p*	True negatives	False positives	*p*
*n*	25	4		129	5	
Age, years	86 ± 5	84 ± 9	0.63	74 ± 14	82 ± 11	0.23
Sex M, %	46	67	0.52	40	17	0.25
Systolic BP, mmHg	123 ± 18	121 ± 14	0.81	127 ± 18	125 ± 25	0.83
Diastolic BP, mmHg	70 ± 12	76 ± 10	0.41	75 ± 16	80 ± 10	0.61
Heart rate, bpm	82 ± 20	82 ± 46	0.96	75 ± 17	80 ± 10	0.52
Upper arm circumference, cm	28 ± 1	28 ± 1	0.95	28 ± 3	27 ± 3	0.66
RR-CV	0.19 ± 0.06	0.09 ± 0.02	< 0.01	0.10 ± 0.09	0.05 ± 0.05	0.25

*BP* blood pressure, *RR-CV* coefficient of variability of 10 consecutive R–R intervals evaluated by the electrocardiogram

When the analysis was restricted to patients aged ≥ 65 years (*n* = 134, 82% of the total population), results did not significantly change (Cohen kappa’s coefficient 0.81, *p* < 0.001, sensitivity 86%, specificity 95%, Table 4). AF signaling was found positive in all patients with new-onset AF (100%).

**Table 4:** Level of agreement (LOA), sensitivity, and specificity of first and second measurements for AF screening, and their combinations

	Kappa	LOA	Sensitivity (%)	Specificity (%)
First reading	0.81**	Almost perfect	86	96
Second reading	0.82**	Almost perfect	79	99
Any positive reading	0.82**	Almost perfect	90	96
Both readings positive	0.82**	Almost perfect	76	99
> 65 years (first reading)	0.81**	Almost perfect	86	95

*Kappa* Cohen’s kappa agreement

**p < 0.01

The AF screening was repeated in all patients; results of repeated measures were concordant with the first evaluation in 153 of 163 individuals (94%); in seven cases, the second screening test did not confirm the positive results at first evaluation, whereas in three cases, that were negative at the first evaluation, the second screening was positive for AF (Cohen kappa’s coefficient 0.82, *p* < 0.001, sensitivity 79%, specificity 99%). The comparison of the two tests did not show any significant difference in terms of sensitivity (*χ*^2^ = 0.80, *p* = 0.37) or specificity (*χ*^2^ = 0.25, *p* = 0.62). When AF signaling was assigned based on positivity of any of the two readings or based on both positive results at the first and second readings, the overall level of agreement did not significantly change.

## Discussion

In our study, we found that in patients routinely admitted to the Internal Medicine ward, in whom we showed a relatively high prevalence of AF episodes (18%), the use of the Microlife WatchBP Office AFIB, a BP oscillometric device enabling AF screening based on the analysis of pulse irregularities during BP cuff deflation was effective in detecting AF. We found an almost perfect level of agreement with AF rhythms simultaneously diagnosed by ECG (Cohen kappa’s coefficients between 0.81 and 0.82, *p* < 0.001), with good, although sub-optimal, sensitivity (ranging from 76 to 86%) and even higher specificity (between 95 and 99%) irrespective of whether AF screening was performed by one or two sets of measurements (each set was constituted by 3 BP successive measurements with an interval of 15 s for each one), or by any of these combinations. Interestingly, we observed that all the new-onset AF episodes, representing the 21% of all AF detection, were correctly identified at the AF screening. Moreover, when we analyzed the sources of discrepancy within groups, we found that false negatives were characterized by ECG traces with an almost regular R–R succession (coefficient of variation between R–R intervals 0.09 ± 0.02).

Our results are generally in line with previous research performed on the same field. Previous studies reported rather similar levels of accuracy of the Microlife WatchBP automated algorithms for AF detection [17–21], although they were recorded in different clinical settings. In many cases, both sensitivity and specificity were found to be superior to pulse palpation (sensitivity 87%, specificity 81%) performed by general practitioners or nurses [22], although the overall performance of AF screening by pulse palpation might be influenced by many factors, including the level of training of the medical staff [23]. Interestingly, our data were almost perfectly superimposable to results reported to another inpatient setting with similar AF prevalence (18%) [24]. Of note, our study was designed to perform simultaneous full 12-lead ECG to all the enrolled patients and not only to those who were screened positive immediately after a possible AF was detected by the device [15, 21, 25, 26], as often done in previous research. This led us the possibility to detect the true number of false-negative AF episodes by avoiding overestimation of sensitivity, which could occur if a negative AF screening would not receive further confirmation by 12-lead ECG diagnosis of non-AF rhythm. We believe that this was one of the main reasons explaining the sub-optimal sensitivity for AF detection found in our study as compared with previous studies on the same issue [21, 25]. For the same reason, we chose to not compare the accuracy of this tool with medical processes usually performed in clinical practice to screen for AF, such as pulse palpation or cardiac auscultation, because ECG is not routinely used for AF screening purposes. It remains to be demonstrated, in the future, if a modified algorithm using, for instance, a stricter cut-off for the detection of irregular heartbeats, could lead to an increase in sensitivity without a parallel decrease in specificity.

Our results related to new-onset AF episodes deserve appropriate discussion. Previous research suggested new-onset AF episodes are frequent during hospitalization, and that the majority of in-hospital new-onset AF episodes is triggered by acute conditions such as infections, septic shock, thyrotoxicosis, hyperglycemia, or pulmonary embolism. Moreover, these episodes are associated with high rates of recurrences and adverse outcomes, including increased in-hospital mortality [7, 9]. Given that these clinical conditions are frequently observed in patients hospitalized at the Internal Medicine wards [8], it is of note that the good performance of the Microlife WatchBP Office AFIB device for new-onset AF screening makes this technology particularly suitable for the routine daily assessment of vital parameters in this clinical setting. Moreover, as opposite to pulse palpation, this screening technology is operator independent, does not require adequate training, and ideally would be highly effective in favouring a prompt AF diagnosis and a timely administration of anticoagulant drugs in patients where indicated, without any additional cost. However, if the routine adoption of this technology would translate into a decrease of death and disability for Internal Medicine inpatients remains to be demonstrated in future dedicated studies.

Our results are limited by a number of factors. First, the main findings of our study, especially those related to new-onset AF, are limited by the small sample size and they need to be confirmed by further studies evaluating larger populations of hospitalized patients. Moreover, considering that sensitivity and specificity of a diagnostic test may vary with disease prevalence, we cannot exclude that results might not be repeatable if performed in a different clinical context characterized by different AF prevalence. Second, although easily repeatable, AF screening performed during automated BP measurement should be considered as a single time point methodology for AF screening, and could be, therefore, underpowered to detect paroxysmal AF episodes or other arrhythmias as compared to methods enabling continuous AF screening (e.g., implantable loop recorders). Third, results obtained with the Microlife WatchBP Office AFIB device in our study cannot be extended to other BP monitors equipped with AF screening algorithm. Comparison between various devices provided different results in previous research [27, 28]. Being such comparison beyond the scope of our article, our results may not be extensible to other devices equipped with different algorithms for AF detection. Finally, we found a very low proportion of arrhythmias other than AF.

In conclusions, our study shows that systematic AF screening during the hospitalization at the Internal Medicine ward performed by the Microlife WatchBP Office AFIB, an automated BP monitor equipped with an algorithm enabling AF screening, is feasible and effective in detecting AF, including new-onset AF episodes. If our results will be confirmed in larger populations, such technology could be readily implemented in current clinical practice to prevent undiagnosed AF episodes, particularly new-onset AF episodes, often complicating the clinical course of hospitalized patients and in doing so, to reduce the overall burden of undetected AF and associated risks of deaths and disability. Further studies are needed to demonstrate if AF screening performed during automated BP measurement would effectively improve AF diagnosis and management and translate into an overall reduction of CV mortality.

## Data Availability

Data from which the results were derived are available upon reasonable request.
